# Evaluation of Implant Placement Risk Levels in Partially Edentulous Patients Using Cone Beam Computed Tomography

**DOI:** 10.7759/cureus.47893

**Published:** 2023-10-29

**Authors:** Sary Borzangy, Ahmed Yaseen Alqutaibi, Majid Krsoum, Rana Aljohani, Osama Qadri

**Affiliations:** 1 Substitutive Dental Sciences, College of Dentistry, Taibah University, Madinah, SAU; 2 Dentistry, King Abdulaziz Medical City, Ministry of National Guard - Health Affairs, Jeddah, SAU; 3 Dentistry, Magrabi Dental Center, Madinah, SAU

**Keywords:** partially edentulous, esthetic, jaw bone anatomy, dental implant, cbct

## Abstract

Objective: This study aimed to evaluate the implant placement risk levels in partially edentulous patients using cone beam computed tomography (CBCT) and implant planning software.

Materials and methods: A total of 101 CBCT scans of partially edentulous patients were included. Evaluations of jawbone anatomy, vital structures' proximity, and the risk of inferior alveolar nerve injury with esthetic considerations were done on CBCT images using implant surgery software (Blue Sky Plan 4; Blue Sky Bio, Grayslake, IL).

Results: A total of 101 patients with 106 edentulous sites were examined. The mean ridge height of the non-esthetic zone was 10 mm and 14.4 mm in the maxilla and mandible, respectively. The period of edentulousness significantly affected the risk of placing implants in the non-esthetic zone (P<.05). The relation between gender and mandibular canal identification was significant (P<.01). A higher risk of implant placement is associated with a more extended period of edentulousness. The preoperative assessment revealed that the mandibular canal could be identified more easily in females than males; thus, nerve injury could be avoided. However, age had no associated effect.

Conclusion: Age has no associated effect on the implant placement risk levels for partially edentulous patients during dental implant planning. A higher risk of implant placement is associated with a more extended period of edentulousness. Mandibular canal identification during virtual preoperative assessment was higher in females.

## Introduction

Tooth loss is a significant concern that affects different age groups and is attributed to several factors, such as caries, trauma, and periodontal disease [1]. Consequently, tooth loss may lead to the depletion of oral functions [2]. Residual ridge resorption is described as bone resorption after tooth extraction; it peaks in the first three months and gradually decreases with time [3]. Local and systemic factors are vital in the resorption process [4].

Dental implant-supported prostheses are widely used to restore missing teeth [5]. However, the placement of a dental implant can be limited in certain conditions, e.g., in a patient who is not fully grown, tooth loss at a young age may complicate the provided treatment plan, particularly, if occurring in the esthetic zone.

Accurate morphologic evaluation of the residual ridge and bone is crucial for prosthetic treatment planning of dental implants [6]. In this regard, cone beam computed tomography (CBCT) enables visualization of bone levels with its three-dimensional imaging technique that provides axial, coronal, and sagittal multi-planner images [7]. CBCT used in dental implant treatment planning can help evaluate alveolar ridge, proximity to vital anatomical structures, and the fabrication of surgical guides [8].

In 2013, a clinical and radiological classification system of the jawbone was proposed by Juodzbalys and Kubilius to help with implant treatment planning [9]. It includes assessing the edentulous jaw segment, the proximity of vital structures, and the risk of inferior alveolar nerve injury with esthetic considerations in endosseous dental implant treatment. All these parameters were classified into Type I (low risk), Type II (moderate risk), and Type III (high risk) [9]. However, the gender, age of patients, and the period of edentulousness were not included in this classification.

Despite the belief that age does not affect residual bone resorption, several studies have reported that a certain amount of alveolar bone loss occurs with aging [10,11]. Moreover, Ikebe et al. recommended finding evidence for risk factors related to aging, especially in the success and survival of the dental implant [12]. Additional research is warranted amidst the contentious nature of the subject matter and the suggested course of action. Therefore, this study aimed to evaluate the surgical and esthetic risk of implant placement in partially edentulous patients in regard to age, gender, alveolar bone height, and period of edentulousness by using CBCT and an implant planning software. The hypothesis was that the surgical and esthetic risk of implant placement in partially edentulous patients has a relation to patients’ age and gender, alveolar bone height, and period of edentulousness.

## Materials and methods

This retrospective study aimed to examine the three-dimensional analysis of the edentulous jaw segment in patients who had received CBCT imaging at Taibah University Dental Hospital (TUDH) during the years 2018 and 2022. This work was approved by the Research Ethical Committee of the College of Dentistry at Taibah University, Madinah, Saudi Arabia (approval no. 20170130). Following verbal consent from the participants, a telephonic questionnaire was administered to gather updated information about their age, overall health, dental status, smoking habits, and history of tooth extraction. Each subject was assigned an anonymous code, and all data was stored in an Excel spreadsheet protected by a security password. Additionally, the computer system was secured with a password.

The study used CBCT images of partly edentulous adult patients aged 18 to 72 years from Saudi Arabia. The study excluded patients with a prior history of trauma, disease (e.g., patients on bisphosphonates and medical conditions that require prolonged use of steroids), surgical intervention, congenital syndrome, an implant, or any other foreign body that caused artifacts in the image around the facial features or any fractures in the edentulous area. Furthermore, images that exhibited blurriness, characterized by a lack of a well-defined and an obvious demarcation of bony boundaries were excluded. Out of a total of 870 scans that were examined, 101 scans met the requirements for eligibility.

The investigators participated in training sessions utilizing the Blue Sky Plan 4 implant surgery software (Blue Sky Bio, Grayslake, IL), designed explicitly for cone beam computed tomography. The investigators analyzed the CBCT images during these sessions to establish a standardized reading and interpretation approach. To ensure efficient data-collection calibration and standardize the reading and interpretation, two researchers with more than 10 years of experience in CBCT analysis and interpretation shared software instructions and repeated appropriate modifications. Each examiner examined 10 CBCT scans before the actual CBCT scan examination; these scans were chosen from extra data and are not included in the findings of this study. The arithmetic average of the two values was considered and incorporated into the statistical analysis.

The CBCT machine, namely, the KaVo 3D eXam model (KaVo) was utilized at the TUDH to acquire all the CBCT scans. The images were acquired using a 120 kilovolts peak (kV) voltage and a 5 milliamperes (mA) current. The field of view (FOV) was set at 16×13 cm, with a voxel size of 0.25 millimeters. The acquisition time for each image was 26.9 seconds. All CBCT scans conducted at the TUDH were executed using a standardized scanning technique.

**Figure 1:**
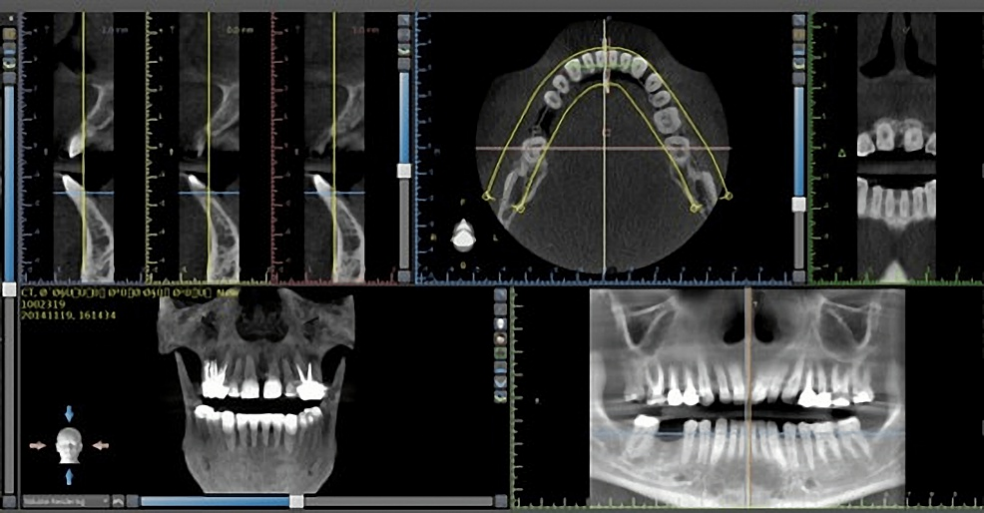
Reconstruction of axial, sagittal, cross-sectional, and panoramic images in Blue Sky software program

The patients were placed in a horizontal posture and given head and chin supports tailored to their specific needs. The patients maintained a state of immobility throughout the duration of the scanning process. The dental arches, specifically the maxilla and mandible, were recreated using sagittal, panoramic, and axial images, as shown in Figure 1.

A total of 101 CBCT scans were analysed, encompassing 106 locations that were devoid of teeth. This study incorporated all the factors outlined in the categorization proposed by Juodzbalys and Kubilius [9], which was deemed necessary to validate. In this study, Straumann standard bone level implants of varying lengths (8, 10, 12, and 14 mm) and diameters (3.3, 4.1, and 4.8 mm) were employed to facilitate the restoration of lost teeth.

As per the guidelines provided by the manufacturer, a diameter of 3.3 mm was not employed in the posterior regions. Various statistical tests were conducted using IBM SPSS Statistics, version 19 (IBM Corp., Armonk, NY) to analyse each aspect of the study objective. In the case of age, which was characterised by normally distributed continuous data, an ANOVA test was employed. The chi-square test was employed to examine the gender variable, which was treated as categorical data. The distribution of continuous data during the period of edentulousness in the aesthetic zone was shown to deviate from normality. Therefore, the Kruskal-Wallis test was employed. The ANOVA test was used to analyse the duration of edentulousness in the non-aesthetic area, as this variable had a normal distribution and was measured on a continuous scale.

## Results

A total of 101 CBCT scans with 106 edentulous sites were examined. Of the 106 edentulous sites, 32 (29.2%) were in the esthetic zone, and 74 (69.8%) were in the non-aesthetic zone. Moreover, 44 (41.5%) were maxillary edentulous sites, and 62 (58.5%) were mandibular edentulous sites. The distribution of the esthetic and non-esthetic zones in both arches is presented in Table 1.

**Table 1:** Distribution of the selected cases in esthetic and non-esthetic zones in both arches

Edentulous jaw sites	Edentulous zones, n (%)		
	Esthetic zone	Non-esthetic zone	Total
Maxillary	21(19.8)	23 (21.7)	44 (41.5)
Mandibular	11 (10.4)	51 (48.1)	62 (58.5)
Total	32 (29.2)	74 (69.8)	106 (100)

The Pearson correlation coefficient was employed to evaluate the degree of agreement between two raters by comparing their respective measurements. The results indicated a high level of agreement between the two raters, with a reliability of 89.4%.

The mean age of patients was 42±13 years. The period of edentulousness ranged from less than a year to 46 years in the esthetic edentulous areas. However, the non-esthetic zone lasted less than a year to 19 years.

The participants were asked about some factors that might correlate to bone loss, i.e., smoking, chronic diseases, periodontal status, and way of extraction. The percentage of smokers was 12% in the esthetic zone and 18% in the non-esthetic zone. Diabetes was seen in 6% in the esthetic zone and 14% in the non-esthetic zone. Periodontal diseases were seen in 31% in the esthetic zone and 35% in the non-esthetic zone. In addition, most of the patients had undergone simple extraction, as found for 90.6% in the esthetic zone and 86.5% in the non-aesthetic zone.

The factors considered to specify the patients' risk for implant placement were height, width, and length of the bone for the esthetic or non-aesthetic zone. The interdental bone peak height for the esthetic zone and mandibular canal identification for the non-esthetic zone were assessed, as presented in Table 2.

**Table 2:** Bone dimensions and planned implant sizes in the selected cases BA, bone augmentation; Ortho, uprighting and orthodontics

Variables		Edentulous jaw segment (mean±SD)			
		Esthetic zone		Non-esthetic zone	
		Maxilla	Mandible	Maxilla	Mandible
Edentulous jaw segment parameters (mean±SD, in mm)					
		15±4.3	14.7±2.4	10±4.6	14.4±2.8
Width		5.7±1.7	5.5±2.3	8.8±2.5	7.9±2.3
Length		6.5±1.2	6.9±1.9	8±3.1	8.5±4.7
Interdental bone peak height	Mesial	4±0.9	4.4±1.3		
	Distal	4±0.9	4.6±1.3		
Size of the endosseous dental implant virtually planned using the Sky Blue, n (%)					
Length of implant	8 mm	7 (33)	2 (18)	13 (57)	7 (14)
	10 mm	4 (19)	1 (9)	7 (30)	19 (37)
	12 mm	9 (43)	8 (73)	-	4 (8)
	14 mm	1 (5)	-	3 (13)	21 (41)
Diameter of the implant	3.3 mm	17 (81)	10 (91)		
	4.1 mm	4 (19)	1 (9)	18 (78)	34 (66.7)
	4.8 mm	-	-	5 (22)	16 (31.4)
Additional procedures	BA	7 (33)	1 (9)	13 (57)	23 (45)
	BA and Ortho	6 (29)	7 (64)	4 (17.4)	5 (10)
	Ortho	7 (33)	-	4 (17.4)	-

Although the mean bone height was classified as low risk in both zones, the shortest mean height was found in the maxillary non-aesthetic zone, and most virtually planned implants were 8 mm in this site. The esthetic zone in this study had medium- or high-risk levels of implant placement, as shown in Table 3. However, the non-esthetic zone in this study had low-, medium-, or high-risk levels of implant placement, as shown in Table 4.

**Table 3:** Different factors in relation to the edentulous jaw segment in the esthetic zone

Variables		Edentulous jaw segment in the esthetic zone, n (%)		
		Low risk	Medium risk	High risk
Gender	Males	-	12 (75)	4 (25)
	Females	-	7 (44)	9 (56)
Diabetic status	Diabetic	-	1 (50)	1 (50)
	Non-diabetic	-	10 (53)	9 (47)
Smoking habit	Smokers	-	2 (50)	2 (50)
	Non-smokers	-	17 (61)	11 (39)
Periodontal diseases	Yes	-	5 (50)	5 (50)
	No	-	14 (64)	9 (36)
Total		-	19 (59)	13 (41)

**Table 4:** Relation of different factors to the edentulous jaw segment in the non-aesthetic zone

Variables		Edentulous jaw segment in the non-esthetic zone, n (%)		
		Low risk	Medium risk	High risk
Gender	Males	15 (56)	7 (26)	5 (18)
	Females	13 (35)	14 (38)	10 (27)
Diabetic status	Diabetic	3 (33)	3 (33)	3 (33)
	Non-diabetic	13 (36)	14 (39)	9 (25)
Smoking habit	Smokers	6 (67)	3 (33)	-
	Non-smokers	22 (40)	18 (32)	15 (27)
Periodontal diseases	Yes	9 (40)	9 (40)	4 (18)
	No	19 (54)	12 (29)	11 (26)
Total		28 (44)	21 (32)	15 (23)

Age was found to have no significant effect on the risk level of implant placement. However, gender substantially related to mandibular canal identification (P<0.01). The females were more likely to have a lower risk of mandibular canal identification for implant placement than the males. Regarding the period of edentulousness, the non-aesthetic zone showed a significant relation to implant placement risk levels (P<0.05).

## Discussion

Evaluating implant placement risk levels of partially edentulous patients in relation to patients' age, gender, and period of edentulousness was this study's primary aim. However, assessing the effectiveness of some chronic diseases on a residual ridge, measuring the proximity of planned implants to vital structures, and determining the required bone graft for planned implants were other objectives. The hypothesis was rejected except for gender that was related to mandibular canal wall identification, and the period of edentulousness for the non-aesthetic zone that demonstrated a significant relation in this study.

Age has no significant relation to implant placement risk levels. This certainty is based on what was reported by Al-Jabrah in 2011, who found no significant difference, between the two groups included, regarding age and edentulousness time; it was also found that mandibular residual ridge resorption was directly related to the duration of edentulousness [13]. Also, Liang et al. reported that age does not affect residual ridge resorption [14].

Gender was significantly related to mandibular canal identification in this study, as the identification of the mandibular canal during virtual implant placement was higher in females. Therefore, inferior alveolar nerve injury complications would be lower in females. This may be due to the demographic nature of the study sample; the analysed records did not include elderly patients (60 years or above), and medical conditions such as osteoporosis or hormonal changes were not detected.

Furthermore, the period of edentulousness significantly affected the risk of placing an implant at an edentulous area in the non-aesthetic zone for the three risk levels. Based on what Atwood reported, residual ridge resorption starts rapidly in the first 6 months to 2 years and might continue until death [15]. Therefore, a shorter period of having no teeth is associated with a lower risk than a longer period requiring additional procedures. However, when it comes to missing teeth that affect appearance, there is no significant correlation between the length of time without teeth and the risk levels of placing implants. In the esthetic zone cases, regardless of the patients' medical condition, all exhibited medium- or high-risk levels for certain risk factors. These findings align with the Straightforward, Advanced, Complex (SAC) classification and the research by Buser et al. [16].

This study found that the maxilla has less alveolar bone height than the mandible in the non-aesthetic zone. A similar finding was reported by a previous study, which showed the maxilla had more resorption than the mandible in relation to local factors [17]. The mandible had more resorption when it came to systemic factors.

When the ridge's height is decreased, insufficient bone may be available to support lengthier implants adequately; as a result, additional surgical treatments may be required [18]. Short implants are typically associated with less complexity and invasiveness than operations involving bone grafting or sinus lift techniques and can avoid or reduce the necessity for these treatments, reducing patient discomfort and healing time [18-21].

In our investigation, CBCT was employed to assess the anatomical differences present in the posterior region of the jaw. The utilization of CBCT technology in dentistry practice has facilitated the process of three-dimensional craniofacial assessment due to its superior image quality, reduced radiation dosage, and lower cost compared to traditional computed tomography [22]. The image resolution is influenced by CBCT parameters. Therefore, all CBCT scans utilized in this study were acquired under identical conditions, employing the same machine and settings (such as scan mode, kilovoltage, voxel size, rotation degree, field of view, milliamperage, and exposure duration). This standardization was implemented to ensure that the visibility of anatomical structures remained unaffected [23]. Due to technological advancements, CBCT offers significant potential for a comprehensive assessment of several maxillofacial areas in multiple planes. This includes the evaluation of dimensions, position, and prevalence of the lingual concavity, as well as assessing bone quality and quantity required for dental implant placement. Using CBCT for estimating anatomical regions in diverse populations can assist operators in customizing treatment planning. This, in turn, facilitates the development of suitable protocols for implant placement, ultimately leading to practical outcomes in implant procedures [24-26].

Several limitations to this study should be considered: the generalizability of the findings may be limited due to the study's exclusive focus on a particular center (TUDH). However, it is essential to consider that the Al Madinah city accommodates a diverse population from many ethnic backgrounds. Another limitation is the relatively small sample size of 101 participants. Increasing the sample size would enhance the representativeness of the results, rendering them more reflective of the broader population.

## Conclusions

Based on the findings of this study, the following conclusions were drawn: first, age was found to have no impact on implant risk levels. Second, the identification of the mandibular canal during virtual preoperative assessment was more successful in females. Last, a longer period of edentulousness was associated with a higher risk of implant placement. These conclusions highlight the importance of determining implant placement risk levels for partially edentulous patients as it can aid in treatment planning and predicting treatment outcomes.

Further research is warranted to examine the anatomical variations in edentulous areas among a large group of diverse ethnic populations worldwide. It is essential to consider the potential influence of various factors, such as body mass index, on the characteristics of these anatomical structures.
